# A Hope-enhancement Instrument for Palliative Care Cancer Patients

**DOI:** 10.7759/cureus.5342

**Published:** 2019-08-07

**Authors:** Yusuf Adnan Guclu

**Affiliations:** 1 Family Medicine, Izmir Health Sciences University, Tepecik Education and Research Hospital, Izmir, TUR

**Keywords:** palliative care, neoplasms, hope, intervention study

## Abstract

Aim

Millions of new cancer cases are diagnosed each year. ‎Patients often become hopeless during the disease. This study aimed to develop a short-intervention instrument targeted to raise hope in cancer patients.

Methods

Using a panel of experts, an instrument was developed, which consisted of 11 counseling items. The instrument was applied to a sample of 153 palliative care cancer patients randomized into three groups (G1: instrument applied by Rater 1, G2: control, and G3: instrument applied by Rater 2). Application of the instrument required 20-30 minutes. Using the Herth Hope Index (HHI) scores as the main outcome, changes over time (baseline, 1-hour, and one-week) were evaluated.

Results

The mean baseline HHI scores were 41.38‎±4.46. ‎The HHI scores were statistically similar at the ‎baseline (p>0.05) but significantly different at one hour and one week in favor of the G1 and G3 groups (p<0.001). In G1, the HHI significantly increased from baseline to one-hour measurements (t=-12.413, ‎p<0.001) and remained unchanged at one week (t=1.088, ‎p=0.282). Similarly, there was a significant increase in the HHI scores from baseline to one-hour ‎measurements in G3 (t=-9.144, p<0.001), which remained unchanged between one hour and one week (t=-0.099, p=0.921).

Conclusion

This study demonstrated the effectiveness of a structured, short counseling intervention in increasing ‎hope among palliative care cancer patients.

## Introduction

If the proverb “Hope is the bread of the poor” would be translated into health, it should probably ‎say “Hope is the remedy of the sick.” Cancer, which is one of today’s leading health problems, is perceived as a serious and chronic disease that causes desperation and uncertainty, evokes pain, reminds death, raises guilt and anxiety, and creates panic and confusion [1]. Current treatment methods of cancer, which are targeted to prolong the duration and quality of life, also bear some shortcomings. Patients often become hopeless during the disease progression and side effects of the treatments‎.

Defined as “an expectation of a personal tomorrow” [2], hope is a dynamic power that endows the individual to adapt and engage with the future and find the meaning of his life [1]. Hope is one of the most vital factors to effectively cope with the loss, uncertainty, and pain caused by cancer. Hope is a vital factor that increases the motivation of the individual and supports the person in coping with feelings of pessimism and helplessness. Also, hope has an important place in the adaptation of cancer patients to their diseases and treatment processes [3]. Thus, hope is an essential coping source, especially for cancer patients. The presence of spiritual resources, family members, health workers, and treatment options was determined as the most crucial factors affecting hope [4].

Research has been conducted for many years to define the prospect and dimensions of hope [1]. One well-known tool in this regard is the Herth Hope Index (HHI) developed by Herth in 1992 [5]. On the other hand, researchers have set strategies to increase hope [6-9]. In this context, Herth has identified seven hope development strategies [9]. These include “Interpersonal connectedness,” “Spiritual base,” Attainable aims,” “Affirmation of worth,” “Lightheartedness,” “Personal attributes,” and “Uplifting memories,” while “Abandonment,” “Uncontrollable pain and discomfort,” and “Devaluation of personhood” were mentioned as hope-hindering categories [10].

A growing number of studies advocate the integration of spirituality with health services. However, there are also researchers who state that research on hope-raising strategies provides evidence of hope and satisfaction in life but that there is limited evidence that it reduces psychological stress. Besides, there is insufficient evidence of what can be done to implement hope-building strategies [8]. In 2001, Kaye Herth developed a package called Hope Intervention Program (HIP) that aims to increase hope [11], where it has been shown that it is possible to produce and maintain hope with appropriate methodology.

It is essential that healthcare providers have guides and instruments to help their patients and to implement strategies to increase hope [3]. It is crucial that beyond who and where the procedures are practiced, the content of these initiatives is standard, and that everyone who is trained is able to implement them. The program content should be applicable in the patient room, at home, in outpatient clinics, in waiting rooms, by telephone, or even by self-reading.

In the palliative care context, it is important to sustain hope but not to give false hope related to the ‎clinical status‎. This study aimed to develop a short-intervention instrument targeted to raise hope and test its effectiveness in cancer patients receiving palliative care.

## Materials and methods

Settings

This instrument-development study was undertaken at the palliative care unit of the Tepecik Education and Research Hospital in Izmir. The unit is on the same campus as the Dr. Suat Seren Chest Diseases Hospital. The unit has a 54-bed capacity and serves patients with different diagnoses by a multi-professional team consisting of family physicians, cancer specialists, nurses, psychologists, religious representatives, social worker, and occupational therapists. The instrument-development and data-collection stages of the study were conducted during March-April 2018 and May-September 2018, respectively.

Study ethics

Ethical approval for the study was obtained from the Atatürk University Medical Faculty local ethics board (IRB number: 3/30, date: 29.03.2018). All participants signed the Informed Consent Form, and the application of the instrument did not involve submitting the patients to any procedure.

Study reporting

The study was conducted and reported according to Brink and Louw‎ [12], who suggested a five-step process: (1) preliminary ‎conceptual decisions, (2) defining key concepts, (3) item generation, (4) assessment of face ‎validity, and (5) formulation of the final tool.

Item generation

The Delphi method [13] was employed during item generation. A team of experts was established, consisting of two family physicians experienced in palliative care, one oncologist, one palliative care nurse, one psychologist, one spiritual care specialist, two palliative care patients, one relative of a palliative care patient, one specialist of geriatrics, and one social service specialist (Total n=11). The Delphi method was applied as two rounds.

Content validity

The researcher asked expert input for two rounds. The Delphi contributors were requested to formulate phrases they use (or suggest to use) to increase hope in cancer patients, which formed a pool of 111 items. Using the inputs of the experts, rephrasing some phrases, and eliminating duplicates, the researcher generated an item pool consisting of 53 Turkish phrases.

The 53 items generated were re-sent to the experts, asking for further suggestions and grading of the items according to relevance, using a five-point Likert scale [14]. A roundtable discussion was conducted with the participation of some Delphi panel members for further refinement and drafting of the instrument, which was concluded on 10 phrases or sentences (Appendix 1). Attention was given by roundtable consensus to include sentences covering all dimensions of hope as described by Herth [9].

Face validity

The instrument was finalized after checking for face validity. For this purpose, the draft instrument was applied to 10 consecutive cancer patients by asking their opinions regarding the general appearance, language, content, and phrasing. The instrument was finalized after a last study team discussion and application of the patient inputs. Any disagreements were discussed at a roundtable, and changes were made to the phrasing and explanation ‎of items.

Population

**Figure 1:**
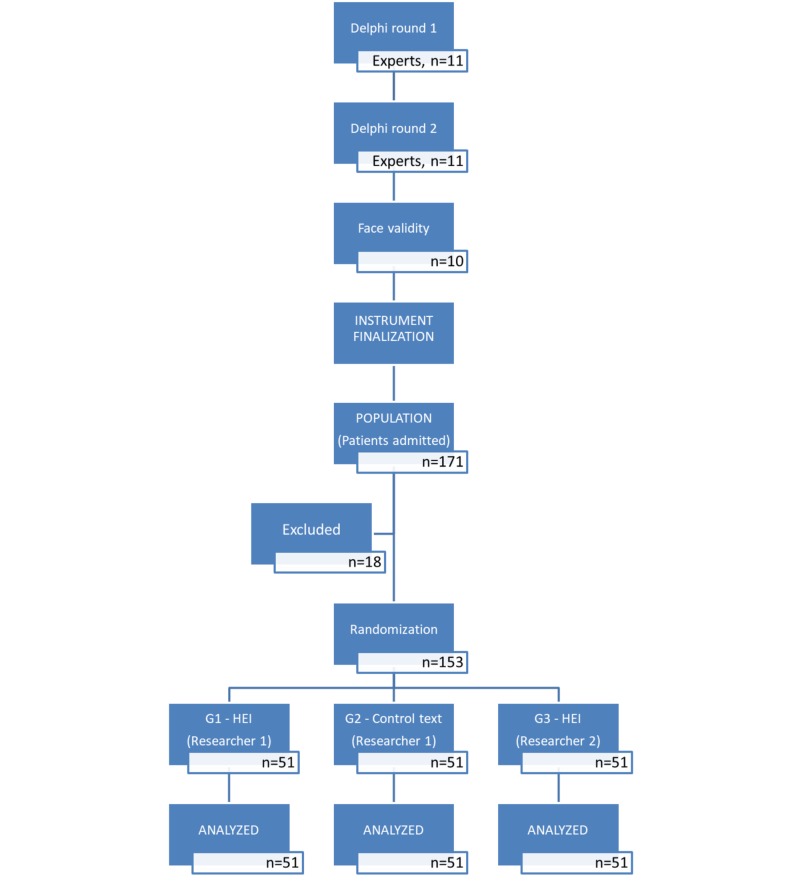
Study flow diagram‎ HEI: Health enhancement instrument.

The population of the study consisted of consecutive patients admitted to the Tepecik Education and Research Hospital palliative care unit during May-September 2018 (n=171, Figure 1).

Study size

Sample size calculation was based on the main outcome HHI score. Forty-nine patients in each group are required to detect a difference of three points in the HHI scores using the independent samples t-test with an effect size of 0.66 (mean 1=43, mean 2=40, standard deviation=4.5), an alpha error of 5%, power of 90%, and a two-tailed hypothesis [15]. Fifty-one patients were included in each group.

Randomization

Consecutive inpatients meeting the inclusion criteria were randomly assigned to either Group 1 (G1; applied the AG Hope Enhancement Instrument (HEI) by Researcher 1), Group 2 (G2; applied the control text by Researcher 1), or Group 3 (G3; applied the Hope Enhancement Instrument by Researcher 2) until the determined sample size was reached (Figure 1).

Sample selection

Patients over the age of 18 admitted to the palliative care unit were included in the study. The following patients were excluded (n=18, Figure 1): Patients having non-cancer diagnoses (n=6), patients who didn’t know their diagnoses (n=4), patients diagnosed for less than one year (n=3), those rejected to give study consent (n=4), and not able to speak Turkish (n=1).‎

Study variables

The main outcome variable of the study was the total HHI score. The Turkish translation of the HHI by Aslan et al. [16] was used for this study. The HHI has 12 items, uses a four-point Likert scale, and can have a total score of 12-48 points. The independent variables studied were ‎age (years), sex (male/female), marital status (single/married/widow/divorced), educational status (illiterate/ primary school/secondary school/high school/license), occupation (retired/housewife/farmer/other), diagnosis (lung/breast/brain/ larynx/stomach/prostate/other), and duration of diagnosis (months).

Data collection

There was no blinding in this study. However, to eliminate researcher bias, data collection was done by the primary author (Researcher 1) and an independent researcher (Researcher 2) (Figure 1). After receiving patient consent, data collection was done in the patients’ room in an anonymous environment. The HEI was applied by the main author (Researcher 1) and another practitioner (Researcher 2) who was instructed on the application of the HEI. Both researchers applied either the HEI or the control text in a friendly environment, reading each passage as described in Appendix 1 and 2, and allowing the patients to reflect and comment. ‎Researchers were allowed to further comment and elaborate each item depending on the case.‎ Demographic data were recorded from the patient files.

First, the HHI was applied, followed by the immediate application of the interventions (either HEI or control text). The researcher returned after approximately one hour to reapply the HHI. The HHI was applied a third time after one week. Application of the HEI to one patient took around 15-20 minutes. A passage related to health from a book (Appendix 2) [17] was modified and read to the control group. Silence and consolidation time were allowed between paragraphs, similar to the hope-enhancement instrument, also requiring 15-20 minutes application time.

Statistical analysis

Data were entered into the computer and analyzed using SPSS 20.0 software (IBM Corp., Armonk, NY, US). Diagnoses with a frequency of less than 10 were grouped under “other.” The results ‎were presented as frequencies, percentages, means, and standard deviation (SD). For the ‎comparison of the demographic and clinical data, the one-way analysis of variance (ANOVA) (with post-hoc Tukey), paired t-test, and independent samples t-tests ‎were used for numerical variables, and the Chi-square test was used for categorical variables. Differences between the three HHI measurements were analyzed with the repeated measures ANOVA test. ‎Associations between various numerical variables were investigated using Pearson's ‎correlation analysis. A p-value of <0.05 was ‎considered statistically significant.‎

## Results

Descriptive data

The study comprised a total of 153 participants (51 patients in each group). The mean age of the participants was 62.66±10.87 (min. 27, max. 88). Most of the participants were men with lung cancer. There were no ‎statistically significant differences concerning the demographic variables of the participants (p>0.05, Table 1).

**Table 1:** Comparison of demographic variables between the groups

Variables	Category	G1 (HEI-Rater 1)		G2 (Control-Rater 1)		G3 (HEI-Rater 2)		p
		n	%	n	%	n	%	
Sex	Male	41	34.2	43	35.8	36	30.0	0.222
	Female	10	30.3	8	24.2	15	45.5	
Primary cancer site	Lung	32	35.2	34	37.4	25	27.5	0.282
	Breast	2	18.2	3	27.3	6	54.5	
	Brain	9	40.9	7	31.8	6	27.3	
	Other	8	27.6	7	24.1	14	48.3	
Educational Status	Illiterate/literate	4	25.0	2	12.5	10	62.5	0.108
	Primary School	40	35.4	40	35.4	33	29.2	
	Secondary School	6	40.0	4	26.7	5	33.3	
	University	1	11.1	5	55.6	3	33.3	
Marital Status	Married	45	34.6	46	35.4	39	30.0	0.371
	Single	1	14.3	1	14.3	5	71.4	
	Divorced	3	37.5	2	25.0	3	37.5	
	Widow	2	25.0	2	25.0	4	50.0	
Occupation	Retired	22	36.7	23	38.3	15	25.0	0.406
	Housewife	11	26.2	12	28.6	19	45.2	
	Farmer	8	34.8	9	39.1	6	26.1	
	Other	10	35.7	7	25.0	11	39.3	

Mean ± SD age in G1, G2, and G3 were 61.75‎±‎9.71, 62.84‎±‎9.95, and 63.39±12.83 years, respectively. Mean ‎±‎ SD time since the diagnosis in G1, G2, and G3 were 25.47±37.26, 16.24±10.97, and 20.37±16.55 months, respectively. There were no differences between the groups concerning age and time since diagnosis (F, p; 0.300, 0.741 and 1.836, 0.163, respectively).

Outcome data

Cronbach’s alpha values for the baseline, one-hour, and one-week HHI scores were calculated as ‎‎0.714, 0.789, and 0.792, respectively.

**Figure 2:**
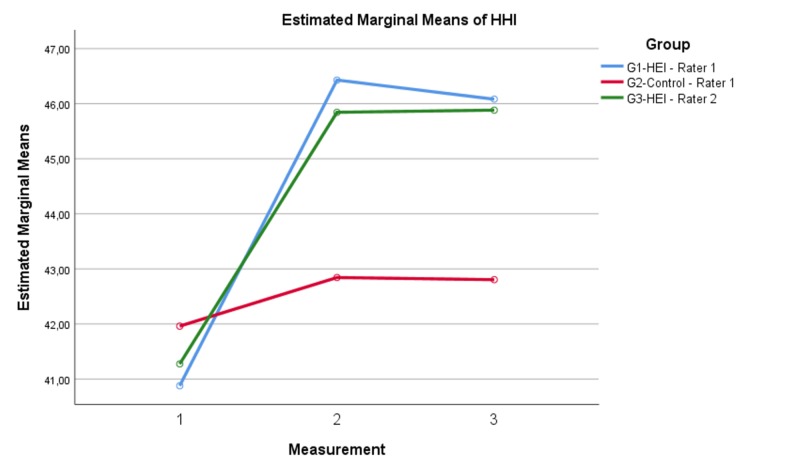
The change in the HHI scores over time HHI: Herth Hope Index; HEI: Hope Enhancement Instrument

The mean baseline HHI scores were 41.38‎±4.46. ‎The HHI scores were statistically similar at the baseline but significantly different at one hour and one week (Table 2, Figure 2). The post-hoc comparisons demonstrated that the differences existed at one hour and one week between the G2 and G1 as well as G3, but there was no difference between G1 and G3 (Table 3).

**Table 2:** Mean HHI scores measured at different time points compared between groups HHI: Herth Hope Index; HEI: Hope Enhancement Instrument

Timepoint	Group	Mean	SD	p
HHI score before the intervention	G1-HEI - Rater 1	40.87	3.73	0.492
	G2-Control - Rater 1	41.96	5.21	
	G3-HEI - Rater 2	41.27	4.39	
HHI score one hour after intervention	G1-HEI - Rater 1	46.42	1.52	<0.001
	G2-Control - Rater 1	42.84	5.24	
	G3-HEI - Rater 2	45.84	2.58	
HHI score one week after intervention	G1-HEI - Rater 1	46.08	2.29	<0.001
	G2-Control - Rater 1	42.80	5.07	
	G3-HEI - Rater 2	45.88	3.18	

**Table 3:** Post-hoc comparisons of HHI scores between groups HHI: Herth Hope Index; HEI: Hope Enhancement Instrument

						95% CI	
				Mean Difference	p	Lower	Upper
HHI score before the intervention							
	G1-HEI - Rater 1		G2-Control - Rater 1	-1.03	0.471	-3.13	1.05
	G1-HEI - Rater 1		G3-HEI - Rater 2	-0.35	0.916	-2.44	1.74
	G2-Control - Rater 1		G3-HEI - Rater 2	0.68	0.719	-1.40	2.78
HHI score one hour after intervention							
	G1-HEI - Rater 1		G2-Control - Rater 1	3.59^*^	<0.001	1.94	5.24
	G3-HEI - Rater 2		G3-HEI - Rater 2	0.59	0.668	-1.05	2.24
	G2-Control - Rater 1		G3-HEI - Rater 2	-3.00^*^	<0.001	-4.64	-1.35
HHI score one week after intervention							
	G1-HEI - Rater 1		G2-Control - Rater 1	3.27^*^	<0.001	1.51	5.03
	G3-HEI - Rater 2		G3-HEI - Rater 2	0.19	0.961	-1.56	1.96
	G2-Control - Rater 1		G3-HEI - Rater 2	-3.07^*^	<0.001	-4.82	-1.33

In G1, the HHI significantly increased from baseline to one-hour measurements (t=-12.413, p<0.001) and remained unchanged even at one week (t=1.088, p=0.282). Similarly, there was a significant increase in HHI scores from baseline to one hour in G3 (t=-9.144, p<0.001), which remained unchanged at one week (t=-0.099, p=0.921). However, although not as marked as in G1 and G3, there was also a significant increase in the HHI scores in G2 from baseline to one hour (t=2.338, p=0.023) and it remained unchanged at one week (t=0.252, p=0.802) (Figure 2).

On the other hand, the differences in the HHI scores between G1 and G3 were not significantly different both at one-hour and one-week measurements (t, p; 1.411, 0.161 and 0.358, 0.721, respectively) (Figure 2).

**Figure 3:**
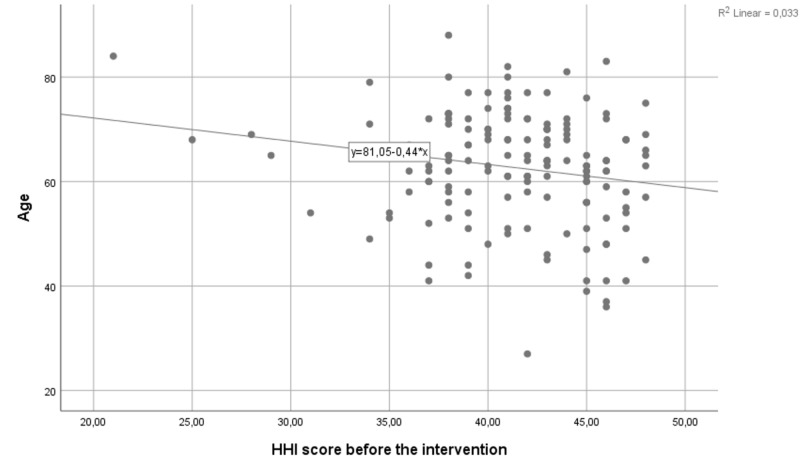
Correlation between age and baseline HHI scores. HHI: Herth Hope Index

There was a weak but significant negative correlation between the patient’s age and baseline HHI scores (Pearson r=-0.182, p=0.024, Figure 3). Also, there was a significant correlation between the baseline HHI scores and scores measured after one-hour and one-week (r, p; 0.607, <0.001 and 0.584,<0.000, respectively).

## Discussion

Key results

This study introduces a short instrument, which proves to be effective in increasing hope in palliative care cancer patients. The proposed AG Hope Enhancement Instrument is easy to apply by health professionals and requires a relatively short time.

Strengths and limitations

Hopelessness is an issue for many patients having non-cancer ailments. Although we considered including patients receiving palliative care due to other conditions, such as stroke, we did not do so for the sake of having a more homogenous set of participants. The only measure used in this study was the HHI. The inclusion of other measures, such as quality of life and wellbeing, would give more ‎information about the impact of the intervention. On the other hand, we had a reasonable sample size and checked for the inter-observer variability of the instrument.

Interpretation

The concept of hope is frequently discussed, and its importance is emphasized in the adaptation to the disease and treatment of patients diagnosed with cancer [4,18-19]. Increasing the awareness of the health care team about the concept of hope, stimulating the patient as a supportive internal factor, and strengthening the patients may increase the coping strategies of the patients in difficult situations.

Cancer is one of the main health problems in the world and its prevalence rising at an alarming rate. Worldwide about 14.1 million new cancer cases and 8.2 million deaths were estimated in 2012 [20]. An extensive review study demonstrated that the most common diagnosis in palliative care units was cancer, followed by end-stage renal disease (ESRD), and chronic obstructive pulmonary disease (COPD) [21]. Although the distribution of cancers in the population [22] follows a trend lung>breast>colorectum, the cases in our sample had a frequency of lung>breast>brain cancers. We think that the reason for the high number of lung cancers in our sample is due to the setting of the palliative care unit. Having close collaboration with a chest hospital nearby, most of the patients referred had lung cancer.

In this study, the mean baseline HHI scores were 41.38. In her original report, Herth calculated the overall mean for the HHI in a mixed group of acute, chronic, and terminally ill patients as ‎32.39 [5]. Aslan et al. [16], the Turkish translators of the HHI reported mean hope values as 38.51 among cancer patients. Afrooz et al. [4] reported the overall score for hope in Iranian cancer patients as 31.4. Using the Beck Hopelessness Scale, Öztunç et al. [23] reported low hopelessness scores in patients with breast cancer. Therefore, the finding of this study highlighted the importance of existing evidence. However, at least empirically, we observed higher hope scores compared with other national and international studies. Although we cannot provide a clear-cut and evidence-based explanation to this difference, the key policy of care at the study center is adding life to the days, not adding days to live. Employing a biopsychosocial and spiritual approach, we collaborate with cancer unions and organize activities and meetings to cheer-up the patients as well as their relatives. Substantial effort is given to ensure that the patients feel in their natural environment in the palliative care unit to the most possible extent. Additional studies are needed to demonstrate whether a real difference in service exists regarding the quality of life and patient outcomes.

The fact that the HHI scores in G2 (control group) were significantly higher at the second measurement can be explained with a placebo effect. Despite the reality that it was initially handled as a nonsense variable, the placebo effect is now recognized as a powerful determinant of health. There are remarkable findings demonstrating that the placebo effect significantly modulates the response to active treatments, especially in painful conditions. It was stated that placebos led to a 35% improvement of symptoms in some conditions [24]. However, having demonstrated a significant difference of increase in the HHI scores after the intervention between both intervention groups (Researchers 1 and 2), we can claim that the hope-enhancement instrument is better than the placebo. Beyond that, the negligible difference between the two raters enables to speak in more confidence about the effectiveness of the new instrument.

The initial increases in the HHI scores were preserved after one week. The intervention we applied in this study may be categorized as a kind of short counseling. Different forms of interventions may have different sizes and durations of effectiveness. Trends towards improvement were demonstrated in the quality of life, uncertainty, depression, and perceived social support with the provision of a psychoeducational intervention in gynecological cancer patients [25]. Cognitive-behavioral therapy was effective in improving tumor-associated fatigue levels in breast cancer patients after eight weeks [26]. A counseling program provided to mastectomy patients produced positive effects upon family functioning and quality of life [27]. Other types of psychosocial interventions, such as case management, general support, and respite, have been shown beneficial in supporting caregivers [28].

The negative correlation between the age and baseline HHI ‎scores suggests that elderly people have less initial hope compared to younger patients, a relationship already confirmed in the literature [29], which disappeared after the intervention.

One fundamental advantage of the AG Hope Enhancement Intervention is probably the relatively short time required for its application. Besides the load to professionals working in palliative care units, the patients may not be suitable for long-term concentration and cooperation. Declines in the cognitive functioning of memory, attention, and executive functions are frequently observed in cancer patients [30]. Hence, a fast and easy-to-apply instrument may fill a gap in the health management needs of palliative care cancer patients.

## Conclusions

This study demonstrated the effectiveness of a structured, short counseling intervention in increasing hope among palliative care cancer patients. Although initial inter-observer reliability could be demonstrated, the instrument has to be tested in other institutions and different health professionals, also checking for its generalizability to cover patients other than cancer. Additional studies are needed to see the long-term effectiveness of this short intervention with the possible contributions of interim boosters.

## Appendices

Dear [Name],

Today, I would like to have a 20-30-minute conversation with you, which, we believe, will contribute to your health. If you accept, I will read you some sentences and get your opinions on them.

**Table 4:** (Appendix 1) The AG Hope Enhancement Instrument (Translated from Turkish).

Item
How are you? You look better today. Although you have a challenging disease process, you are very strong. We really appreciate your determination. It's amazing that you're making such efforts to get better. Keeping your spirits high in this process is the most beautiful thing you can do for yourself. Because the biggest medicine is hope. What do you think?
The patient is given 2-3 minutes to express his / her opinions. If the interview gets too long, the service provider may interfere by saying “Now, I want to read another sentence about the subject,” and proceed to the next item.
Health professionals are there to support you. Trust them;‎ they will be happy if they can help you. What do you think?
The patient is given 2-3 minutes to express his / her opinions. If the interview gets too long, the service ‎provider may interfere by saying “Now, I want to read another sentence about the subject”, and proceed to ‎the next item.‎
Look at the people around you, they all work to encourage you and increase your hope. Besides, you're not the only one who has this disease. People with similar diseases have support groups. In these groups, you can find people to share your troubles. You can join them. What do you think?
The patient is given 2-3 minutes to express his / her opinions. If the interview gets too long, the service ‎‎provider may interfere by saying “Now, I want to read another sentence about the subject,” and proceed to ‎‎the next item.‎
Every challenge is an exam, we must fight to achieve. We believe that you will overcome this exam in the best way. You must have experienced many difficulties until now. What so many difficulties have you overcome… Like all the troubles in the past, we believe you will also get through this one. What do you think?
The patient is given 2-3 minutes to express his / her opinions. If the interview gets too long, the ‎service ‎provider may interfere by saying “Now, I want to read another sentence about the subject,” and ‎proceed to ‎the next item.‎
Medicine is a science that is constantly evolving, new methods and treatments are identified each day. Therefore, new developments can give positive results in your treatment program. Trust in medicine. It is not possible to know exactly how diseases will progress. We have seen that many of our severe patients have recovered; so can you. What do you think?
The patient is given 2-3 minutes to express his / her opinions. If the interview gets too long, the ‎service ‎provider may interfere by saying “Now, I want to read another sentence about the subject,” and ‎proceed to ‎the next item.‎
Everybody has some good memories. I'm sure you have memories you'd like to remember and share. Can you tell me about your memories?
The patient is given 2-3 minutes to express his / her opinions. If the interview gets too long, the ‎service ‎provider may interfere by saying “Now, I want to read another sentence about the subject,” and ‎proceed to ‎the next item.‎
The most important step in a treatment process is to really want to be cured. I congratulate you for being here and taking this step. “No wind can help a ship that does not have a destination”. Every night ends up with a day. We are going to get through these hard times together. What do you think?
The patient is given 2-3 minutes to express his / her opinions. If the interview gets too long, the ‎service ‎provider may interfere by saying “Now, I want to read another sentence about the subject,” and ‎proceed to ‎the next item.‎
I am sure you have dreams, future plans, and things you want to do. Thinking and discussing your plans may contribute to your improvement. Can we speak about your plans and dreams?
The patient is given 2-3 minutes to express his / her opinions. If the interview gets too long, the ‎service ‎provider may interfere by saying “Now, I want to read another sentence about the subject,” and ‎proceed to ‎the next item.‎
There is no room for despair in our culture. Keep your faith high. Your improvement will accelerate as long as you protect your faith. There is a saying “When the whole world wants you to give up, hope whispers: “Try again!” What do you think?
The patient is given 2-3 minutes to express his / her opinions. If the interview gets too long, the ‎service ‎provider may interfere by saying “Now, I want to read another sentence about the subject,” and ‎proceed to ‎the next item.‎
Keeping your daily activities and work can positively impact your treatment. Is there anything you want to do? What can we do for you today within our possibilities?
The patient is given 2-3 minutes to express his / her opinions. If the interview gets too long, the ‎service ‎provider may interfere by saying “Thank you for the discussion. We may continue another time,” and close the session.‎

Dear [Name],

Today, I would like to have a 20-30-minute conversation with you, which, we believe, will contribute to your health. If ‎you accept, I will read you some sentences and get your opinions on them.‎

**Table 5:** (Appendix 2) The text read to the control group

Item
According to a study published in 2004, the first four diseases that cause the most deaths in the US have been reported as Heart Diseases, Cancer, Stroke and Chronic Lower Respiratory Tract Diseases. The causes of death are similar in Turkey. According to research, the first four diseases in Turkey with the highest mortality are ischemic heart disease (21.7%), cerebrovascular disease (15.0%), Chronic Obstructive Pulmonary Disease (5.8%) and perinatal reasons (5.8%). What do you think about these diseases?
The patient is given 2-3 minutes to express his / her opinions. If the interview gets too long, the ‎service ‎provider ‎may interfere by saying “Now, I want to read another sentence about the subject,” and ‎proceed to ‎the next item.‎
The investigators have also studied the real causes behind the diseases with the highest mortalities. Accordingly, the principal reasons leading to more than 40% of deaths are tobacco use, malnutrition/immobility, and alcohol consumption. What do you think?
The patient is given 2-3 minutes to express his / her opinions. If the interview gets too long, the ‎service ‎provider ‎may interfere by saying “Now, I want to read another sentence about the subject,” and ‎proceed to ‎the next item.‎
It is clear that we have to focus on smoking, alcohol, nutrition, and exercise to ensure healthy lives and disease prevention. However, we can see that protection from these health risks is not as easy in daily practice. To avoid these risks, there is no vaccine, medicine, or another remedy. On the contrary, these risks are the result of many intertwined, interrelated factors such as social and cultural norms, technological developments, industry, world order, habits, and motivations. What do you think?
The patient is given 2-3 minutes to express his / her opinions. If the interview gets too long, the ‎service ‎provider ‎may interfere by saying “Now, I want to read another sentence about the subject,” and ‎proceed to ‎the next item.‎
Health care workers, managers, and policymakers who provide individual services to society also have important duties in these matters. How do you think those responsible can accomplish their duties?
The patient is given 2-3 minutes to express his / her opinions. If the interview gets too long, the ‎service ‎provider ‎may interfere by saying “Now, I want to read another sentence about the subject,” and ‎proceed to ‎the next item.‎
Obesity, which is associated with many diseases, has become a worldwide epidemic. What do you think about obesity?
The patient is given 2-3 minutes to express his / her opinions. If the interview gets too long, the ‎service ‎provider ‎may interfere by saying “Thank you for the discussion. We may continue another time,” and close the session.‎
